# Very Important to Physicians

**Published:** 1908-04

**Authors:** G. B. Foscue

**Affiliations:** Secretary Texas State Board of Medical Examiners; Waco, Texas


### Correspondence.

### Very Important to Physicians.

WACO, TEXAS, March 2, 1908.

*Dr. F. E. Daniel, Austin, Texas:*

DEAR DOCTOR: Replying to yours of the 25th, I beg to advise that we herewith enclose the pamphlet, as per your request. Will state, however, that this pamphlet is not satisfactory, from the fact that it is a little misleading, and suggest that you state through your columns that all physicians practicing medicine in Texas, who were legal practitioners under previous laws, are entitled to verification license from this board when satisfactory evidence is produced to that effect.

Those who were practicing medicine prior to January 1, 1885, are required to send their sworn statement to that effect and the affidavit of at least two reputable citizens, corroborating same.

Those whose right to practice is based upon a diploma registered in the district clerk's office in this State subsequent to January 1, 1885, and prior to January 1, 1891, are required to send a certificate from district clerk, showing when and where diploma was issued and when and where same was recorded in this State.

Those whose rights are based upon a diploma recorded in this State from the 1st of January, 1891, to July 9, 1901, are required to send certificate showing when and where same was recorded and when and where issued. Also a certificate from the dean of the college from which they graduated.

Those whose rights are based upon a district board certificate may send either the original certificate or a certified copy of same, and a certificate from the district clerk showing same was recorded prior to the 9th day of July, 1901, or a certificate from the district clerk, showing that judicial district board certificate was recorded prior to July 9, 1901, and also showing what board issued same and when and whether or not same was permanent or temporary.

Those holding State board certificates under the law of 1901 are required to send original certificate, or a certified copy of same, with a district clerk's certificate, showing when and where recorded, and that it was recorded in district clerk's office in this State before the 13th day of July, 1907. Or they can send a district clerk's certificate, showing when and where State board certificate was obtained and when and where recorded. (The latter are verified by books of old boards.)

I trust that I have made this plain and clear to you, and, of course, you will use your own discretion about publishing all or part of same. The trouble with the pamphlets that we sent out is that it does not impress upon applicants the necessity of their district clerk's certificates showing when recorded. Consequently, we have had much trouble from this source.

We are having a good deal of trouble from irregularities of old district board certificates and also same from Eclectic and Homeopathic boards; but we are endeavoring to clear these matters up as rapidly as possible, and have, up to date, verified about thirty-five hundred physicians.

Would be glad if you would note in your Journal that the next regular examination will be held at this place, on the 30th of June, and if you desire it, will send you a copy of our college requirements, for publication. Would be glad in any event to have your criticism of same. I remain, yours very truly,

G. B. Foscue,
Secretary Texas State Board of Medical Examiners.

